# Assessment of equine intestinal epithelial junctional complexes and barrier permeability using a monolayer culture system

**DOI:** 10.3389/fvets.2024.1455262

**Published:** 2024-10-22

**Authors:** Amy Stieler Stewart, Jamie J. Kopper, Caroline McKinney-Aguirre, Brittany Veerasamy, Dipak Kumar Sahoo, John M. Freund, Liara M. Gonzalez

**Affiliations:** ^1^Department of Molecular Biomedical Sciences, College of Veterinary Medicine, North Carolina State University, Raleigh, NC, United States; ^2^Department of Veterinary Clinical Sciences, College of Veterinary Medicine, Iowa State University, Ames, IA, United States; ^3^Department of Clinical Sciences, College of Veterinary Medicine, North Carolina State University, Raleigh, NC, United States

**Keywords:** claudin, horse, jejunum, occludin, scratch assay, transepithelial resistance

## Abstract

Gastrointestinal disease is a leading cause of death in mature horses. A lack of *in vitro* modeling has impeded the development of novel therapeutics. The objectives of this study were to develop and further characterize a small intestinal monolayer cell culture derived from equine jejunum including establishing normal measurements of intestinal permeability and restitution. Three-dimensional enteroids, derived from postmortem sampling of equine jejunum, were utilized to develop confluent epithelial monolayers. The presence of differentiated intestinal epithelial cell types and tight junctions were confirmed using histology, reverse transcription PCR (RT-PCR), RNAscope, protein immunofluorescence and transmission electron microscopy. Transepithelial resistance (TER) and macromolecule flux were assessed as measurements of paracellular and transcellular permeability. Scratch assays were utilized to model and assess intestinal restitution. Monolayer cell cultures reached 100% confluency by ~5–7 days. Equine jejunum monolayers were confirmed as epithelial in origin, with identification of differentiated intestinal epithelial cell types and evidence of tight junction proteins. Function of the intestinal barrier was supported by acquisition of physiologically normal TER values (179.9 ± 33.7 ohms*cm^2^) and limited macromolecule flux (22 ± 8.8% at 60 min). Additionally, following a scratch wound, epithelial cell monolayers migrated to close gap defects within 24 h. In conclusion, this study describes the development of a novel intestinal epithelial monolayer cell culture for equine jejunum, and provides evidence of intestinal epithelial cell differentiation, formation of physiologically relevant barrier function and use as a model of intestinal restitution to test potential therapeutics for equine colic.

## 1 Introduction

Gastrointestinal (GI) disease is the leading cause of death in mature horses (1–3). One contributing factor for the high morbidity and mortality is the disruption of the intestinal mucosal barrier. The intestinal mucosal barrier is formed by columnar epithelial cells connected via tight junctions and serves to guard against translocation of harmful luminal bacteria and associated toxins such as lipopolysaccharide (LPS) (4, 5). Many common causes of colic, such as ischemic lesions of the intestine (i.e., strangulating lipoma, large colon volvulus) and inflammatory conditions (i.e., colitis), result in disruptions to the intestinal mucosal barrier. Thus, treatments that improve intestinal mucosal healing or decrease mucosal damage are desperately needed. The absence of well characterized pre-clinical models to study equine intestinal barrier function is a rate-limiting step in translational research (6). The use of *in vitro* intestinal epithelial cell culture has proven to be a valuable tool in the study of GI disease in a variety of species. Several utilities of these culture systems include the evaluation of candidate treatments to protect and improve intestinal barrier function, acquisition of *in vitro* data regarding intestinal absorption of pharmaceuticals and enhanced understanding of intestinal epithelial cell interactions with microorganisms and intracellular trafficking (7–9).

Confluent intestinal monolayer cell cultures have been developed in other species (5, 10–13). Historically, two methods have been used to develop confluent intestinal epithelial monolayer cell cultures (14): development of a monolayer from primary intestinal cells (11, 12) or from organoids (9, 11, 14, 15). Culture and characterization of 3D small intestinal epithelial cells (i.e., enteroids) have been described in the horse (16–19). Therefore, the objective of this study was to further investigate the barrier function of an epithelial monolayer derived from equine jejunum. Our aim was to successfully grow confluent monolayers that represented the luminal lining found in normal horses and contained multiple relevant intestinal epithelial cell types and tight junctional proteins. Furthermore, we aimed to demonstrate physiologic function through the assessment of intestinal permeability (transepithelial resistance and macromolecule flux) and restitution time (scratch assay). We hypothesized that the cultured intestinal epithelial cell monolayer would differentiate into relevant cell types, develop tight junctions, and recapitulate *in vivo* barrier function properties.

## 2 Materials and methods

### 2.1 Animal and sample collection

This study was reviewed and approved by Iowa State University’s and North Carolina State University’s Animal Care and Use Committees. Tissues were obtained from 5 various breeds of horses that were subjected to euthanasia for reasons unrelated to this study and did not have clinical evidence of GI disease. Horses ranged from 6 to 20 years of age. Immediately following euthanasia, several 20 cm sections of mid-jejunum were obtained through a midline celiotomy and placed in phosphate buffered saline (PBS) on ice for transportation to the laboratory for crypt isolation.

### 2.2 Crypt isolation and enteroid culture

Intestinal crypts from the jejunum were harvested as previously described (17). Enteroid growth was assessed daily. Growth factors were added to the media 48 h (h) after plating and at subsequent 48 h intervals. The entire volume of media was changed every 96 h. Media and growth factors used for enteroid and monolayer cultures (2.3) are described in Table 1.

**Table 1:** Media used for enteroid and monolayer cell cultures.

Media component	Source	Enteroid (2.3.1)	Monolayer (2.3.1)	Monolayer differentiation (2.3.2)
Advanced DMEM	Thermo Fisher Scientific, Waltham, MA	+	+	+
1X N2	Life Technologies Corporation, Carlsbad CA	+	+	+
1X B27 without vitamin A	Life Technologies Corporation, Carlsbad, CA	+	+	+
10 mM HEPES	Life Technologies Corporation, Carlsbad, CA	+	+	+
2 mM glutamax	Life Technologies Corporation, Carlsbad, CA	+	+	+
1X antibiotic-antimycotic	Life Technologies Corporation, Carlsbad, CA	+	+	+
100 ng/mL recombinant Noggin	Millipore Sigma, St. Louis, MO	+	+	+
500 ng/mL recombinant human R-spondin	Sigma Aldrich, St. Louis MO	+	+	+
50 ng/mL recombinant human EGF	Life Technologies Corporation, Carlsbad CA	+	+	+
100 ng/mL recombinant human Wnt3a	R&D Systems Inc. Minneapolis MN	+	+	−
10 mmol/L Y-27632	Sigma-Aldrich, St. Louis, MO	+	+	−
10 mmol/L SB202190	Sigma Aldrich, St. Louis, MO	+	+	−
500 mmol/L LY2157299	Selleck Chemicals, Houston, TX	+	+	+
2.5 μmol/L CHIR99201	Sigma Aldrich, St. Louis MO	+	+	+
500 nM A83-01	Tocris, Minneapolis, MN	+	+	+
0.01 μM [Leu15]-Gastrin I human	Sigma Aldrich, St. Louis MO	+	+	+
0.01 M nicotinamide	Sigma Aldrich, St. Louis MO	+	+	−

+ indicates the presence of the additive in the media used, − indicates the absence of the additive.

### 2.3 Monolayer culture

#### 2.3.1 Standard monolayer subculture

**Figure 1:**
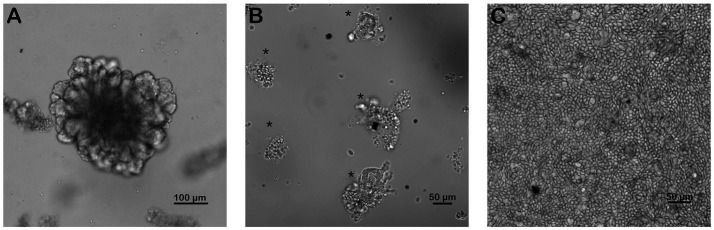
Successful culture of an equine intestinal epithelial monolayer. Mature small intestinal enteroids **(A)** were grown in culture for 5–7 days. Enteroids were dissociated and split into fragments (**B**, *) and replated onto coated transwells. Confluent intestinal epithelial monolayers **(C)** were achieved in 5–7 days. Scale bar, as indicated. *N* = 4.

Monolayers were subcultured from mature enteroids (Figure 1A) on 4.0 μm pore size 24-well transwell plates (Corning 3470, Glendale AZ). First, transwell inserts were coated in 0.5% v/v Matrigel (BD Bioscience (BD), San Jose, CA) in PBS by pipetting 100 μL of 0.5% v/v Matrigel into each well and gently rocking the plate to distribute the Matrigel uniformly over the insert. Plates were then incubated at 37°C for 1 h to allow the matrigel to polymerize. After 5–7 days in culture, mature enteroids were harvested by depolymerizing the Matrigel matrix as previously described (17). After harvesting the enteroids, they were centrifuged at 200–300 G for 5 min, media removed and the resulting pellet was resuspended in 100 μL of advanced DMEM/F12 media (Thermo Fisher Scientific (TFS), Waltham, MA). Resuspended enteroids were vigorously pipetted using 100 and 10 μL pipette tips to dissociate the cells. Cells were counted and subsequently plated at a density of 40–50,000 cells per transwell which took approximately 750–1,000 enteroid fragments per well. Wells were monitored daily and supplemented with media as described for enteroids (2.2) (17).

#### 2.3.2 Evaluation of the effect of growth and differentiation factors on epithelial permeability

In a separate experiment to evaluate the potential effect of differentiation factors on transepithelial resistance, monolayers were identically derived from enteroids and plated in 4.0 μm pore sized transwells (Corning). After 48 h in the same media and growth factors used for previous experiments (17), half of the monolayers were changed to a differentiation-inducing growth factor combination as described in Table 1. Media and growth factors were replaced every other day until the end of the experiment.

#### 2.3.3 Visual confluency assessment

All monolayers were assessed daily using an inverted microscope at 10× and 20× for estimated percentage visual confluency and recorded. Once 100% visual confluency was achieved monolayers were used for further analysis as described below.

### 2.4 RNA extraction and cDNA synthesis

Total RNA was extracted from intestinal crypts, enteroids and confluent monolayers using the Qiagen RNEasy Minikit (Qiagen, Valencia, CA) according to the manufacturer’s recommendations. RNA quality and quantity were assessed by measuring absorbance at 260 and 230 nm. Approximately 125 ng of RNA was utilized to synthesize cDNA using the iScript cDNA Synthesis kit (Bio-Rad, Hercules CA) according to the manufacturer’s recommendations.

### 2.5 RT-PCR analysis for epithelial cell types and tight junction proteins

#### 2.5.1 Epithelial cell types

cDNA synthesized above (2.4) from crypts, enteroids and monolayers were assessed for transcripts from the following epithelial cell types: active stem cells (Sox9, LGR5), reserve stem cell (HOPX, LRIG), epithelial cells (EPCAM), absorptive enterocytes (SIM), goblet cells (MUC2), enteroendocrine cells (CGA), Paneth cells (LYZ) and proliferating cells (PCNA) by RT-PCR using previously published primers and protocols (17). All RT-PCR reactions included a house-keeping gene (*β*-actin) and a no template control. Primers utilized for this study are provided in Table 2. RT-PCR amplicons were assessed on a 2% agarose in tris-EDTA (TE) to evaluate the presence of and confirm amplicon size.

**Table 2:** RT-PCR primer sequences.

	Forward (5′–3′)	Reverse (5′–3′)	Expected amplicon size (base pairs)
Active stem cells			
SOX-9 (17)	AACAAGCCTCACGTCAAGCG	TCCGCCTCCTCCACGAAA	176
LGR5 (17)	AGCCTGGTGGTTCTGCATCT	AACGCTTTCTCGGGGATCAG	200
Reserve stem cells			
HOPX (17)	AGTGGCGGCGGTCGGAA	ACAGTGGCACATACAACACT	167
LRIG (17)	GGCTGACACAACTGGACCTC	TGGTGCCCGAAATGTCGTTA	477
Epithelial cells—EPCAM (17)	TGACCACAAACTGCTCTGTGA	AGCCCGTCGTTATTCTGGAT	180
Absorptive enterocytes—SIM (17)	GCAATACTGGGGGAAGCAGT	CAGATCCAGCAAAAGTTGACC	137
Enteroendocrine cells—CGA (17)	CTGCGAGGAGATGAACGGAT	AGAACCTCTGCGAGTTCGTC	143
Paneth cells—LYZ (17)	CCTGGTCAGCCTAGAGGTCT	TGGCCAAACAGACCCAGTTT	186
Proliferating cells—PCNA (17)	CATGGACTCGTCCCACGTC	CTTCAGCCCTTAGGGTAATG	164
Tight junction proteins			
ZO-1 (20)	CATAGAATAGACTCCCCTGG	CTGCTGGCTTGTTTCTCTAC	190, 430
Occludin (21)	TCTCAGCCAGCGTATTCTTTC	GCACATCACGATAACGAGCAT	111
Claudin-1 (17)	CAGATATGAATTTGGTCAGGCTC	CACTGGAAGGCGAAGGTTT	149
Quality control			
β-actin	CTGTGGCATCCACGAAACTA	GACAATGAGGCCAGAATGGA	237

#### 2.5.2 Tight junction protein

cDNA from crypts, enteroids and monolayers were assessed for the following tight junction proteins: ZO-1 (20), occludin and claudin-1 (21) using previously described RT-PCR protocols. RT-PCR amplicons were assessed on a 2% agarose in TE to evaluate the presence and size of the amplicon. All reactions included a no template control.

### 2.6 Monolayer cell culture fixation for histology and staining

Conditioned media was removed from transwell plates followed by gentle washing of each transmembrane well with 37°C PBS three times. Subsequently monolayers were incubated in 10% formalin at room temperature (RT) overnight followed by storage at RT in 70% ethanol for histology. Fixed cell cultures were paraffin embedded and cut in 4–8 μm sections onto glass slides. For histology, sections were stained using hematoxylin and eosin (H&E) and alcian blue (mucin).

### 2.7 Immunofluorescence analysis for epithelial markers

Following monolayer fixation and slide preparation, deparaffinization was performed using a modified protocol with xylene, ethanol and peroxidase. Heat induced epitope retrieval was performed to improve antigen detection, by heating slides in Reveal Decloaker solution (Biocare Medical, Pacheco, CA) to 120°C for 30 s and then 90°C for 10 s inside a pressure cooker (22). Slides were allowed to cool to RT for 20 min prior to staining. Monolayers were permeabilized in a 0.3% Triton X-100 PBS solution (TTFS) for 20 min and then blocked in protein block (Dako, Carpinteria, CA) solution for 30 min. Primary antibodies were applied in an antibody diluent (Dako) and incubated overnight at 4°C. Dilutions for antibodies included: αB-catenin (mouse, 1:250, Cell Signaling Technology, Danvers, MA) and αE-cadherin (rabbit, 1:250, Abcam, Waltham, MA). All secondary staining was performed with Cy3 and Alexa488 conjugated antibodies diluted 1:500 in antibody diluent incubated at RT for 45 min. Nuclei were marked with bisbenzamide Hoechst 33258 nuclear stain (TFS) diluted 1:1,000 in PBS and applied for 5 min at RT. Background staining was negligible as determined by nonspecific IgG staining. Images were captured on an inverted fluorescence microscope fitted with a monochrome digital camera and color camera. The objective lenses used were X10, X20 and X40 with numerical apertures of 0.3, 0.45 and 0.6, respectively.

### 2.8 Transmission electron microscopy

The equine jejunal monolayers were subjected to fixation for 48 h at 4°C in 0.1 M sodium cacodylate buffer (pH 7.2) containing 1% paraformaldehyde and 3% glutaraldehyde, before being processed for transmission electron microscopy (TEM). Following three washes with 0.1 M cacodylate buffer (pH 7.2), the monolayers were subsequently subjected to a post-fixation process which involved exposing the samples to 1% osmium tetroxide in 0.1 M sodium cacodylate buffer at RT for 1 h. Subsequently, the samples were washed with deionized water and subjected to staining using 2% uranyl acetate for 1 h. A series of dehydration steps were subsequently carried out, wherein monolayer samples were subjected to graded concentrations of ethanol (25, 50, 70, 85, 95, and 100%) for a duration of 1 h each. The samples then underwent further dehydration using pure acetone, with three successive changes, each lasting 15 min. Finally, samples were subjected to infiltration using EmBed 812 formula (hard) EPON epoxy resin (Electron Microscopy Sciences, Hatfield, PA). The infiltration process involved using graded ratios of resin to acetone (3:1, 1:1, 1:3, and pure resin), ensuring complete infiltration with pure epoxy resin, with each step lasting between 6 to 12 h. The tissue samples were carefully inserted into BEEM embedding capsules and subjected to polymerization at 70°C for 48 h. Using a Leica UC6 ultramicrotome (Leica Microsystems, Buffalo Grove, IL), 1.5 μm semi-thin sections were obtained and stained with EMS Epoxy stain (composed of toluidine blue-O and basic fuchsin). Thin sections (50 nm thickness) were prepared and collected onto single-slot carbon film grids, and TEM images were acquired using a 200 kV JEOL JSM 2100 scanning transmission electron microscope (Japan Electron Optics Laboratories, Peabody, MA) with a GATAN OneView 4 k × 4 k camera (Gatan Inc., Pleasanton, CA).

### 2.9 RNAscope multiplex fluorescent RNA *in situ* hybridization assay

For conducting RNAscope *in situ* hybridization (ISH) in formalin-fixed paraffin-embedded (FFPE) monolayer sections, RNAscope Multiplex Fluorescent Kit v2 (ACD Bio, Newark, CA) was employed. RNAscope assay facilitates the detection and estimation of individual RNA molecules, yielding valuable insights into spatial gene expression patterns. Probes to assess occludin, claudin 1, and tight junction protein 1 transcripts in equine jejunal monolayer sections were designed and obtained from ACD Bio (Table 3). Following the manufacturer’s instructions, probes were hybridized to specific tight junction protein marker targets, and specific signals were developed using Opal fluorophores (PerkinElmer Inc., Waltham, MA). For occludin, claudin 1, and tight junction protein 1, Opal 520 (green fluorophore), Opal 570 (orange fluorophore), and Opal 620 (red fluorophore) were used, respectively. Finally, fluorescent signal detection and image acquisition were conducted using a Stellaris STED super-resolution/confocal microscope system (Leica Microsystems Inc., Deerfield, IL), employing Opal dyes with DAPI excitation and emission maxima as recommended by the manufacturer.^1^

**Table 3:** List of RNAscope probes.

Target marker	Target description	Species	Probe	Target region	Reference
Occludin (OCLN)	OCLN, transcript variant X4, mRNA	*Equus caballus*	Ec-OCLN-C1	314–1,312 of XM_023618240.1	1153761-C1
Claudin 1 (CLDN1)	CLDN1, mRNA	*Equus caballus*	Ec-CLDN1-C2	118–1,495 of XM_001500088.4	1153751-C2
Tight junction protein 1 (TJP1)	TJP1, transcript variant X7, mRNA	*Equus caballus*	Ec-TJP1-C3	307–1,283 of XM_023651568.1	1153741-C3

The probes utilized for RNA *in situ* hybridization were obtained from ACD Bio (Newark, CA).

### 2.10 Transepithelial resistance

Transepithelial resistance (TER) was assessed using a Millipore Micell ERS-2 epithelial volt-ohm meter (MS) every 2–3 days once cell cultures approached visual confluency until TER values began to decrease at which time the experiment was terminated. The individual monolayer TER measurements were normalized by comparison to a cell-free insert and then multiplied by the area of the insert (0.33cm^2^).

For comparisons of the impact of different growth factor combinations on TER in monolayers, TER was measured using a STX4 EVOM Electrode (World Precision Instruments, Sarasota, FL), every day from the second day following monolayer plating until the TER decreased for two consecutive days, at which time the experiment was discontinued. The TER measurements were normalized as above.

### 2.11 Measurement of macromolecule (FITC-Dextran 4000) flux

To assess macromolecule flux, once TER values were consistent with *ex vivo* equine jejunum TER values (23–25) and stable the supplemented DMEM/12 media was gently removed and the cell culture washed three times with 37°C PBS. Then, 500 μL of PBS was added to the apical compartment and 800 μL of PBS was added to the basolateral side followed by 10 μL of FITC-Dextran 4000 (50 mg/mL) to the apical side. One hundred μL of media was sampled from the apical and basolateral sides at 0, 30 min and 60 min. Matrigel-coated wells were used as no-cell culture controls. FITC-Dextran 400 concentrations were measured at each time point using a SpectraMax M2e microplate reader (Molecular Devices, San Jose, CA) at excitation and emission wavelengths of 490 and 520 nm respectively.

### 2.12 Scratch assay as a measure of intestinal restitution

To assess epithelial restitution and cell migration, a scratch-wound assay was performed (26). Briefly, once the epithelial monolayers (subcultured, plated and incubated as described in section 2.3.1) had reached 100% confluence, a 200 μL pipette tip was used to scratch a wound evenly through the center of each well. Wells were then washed with PBS and fresh media applied. Scratched monolayers were subsequently monitored and imaged every 6 h to track cell migration.

## 3 Results

### 3.1 Visual confluency was obtained in equine intestinal monolayer

**Figure 2:**
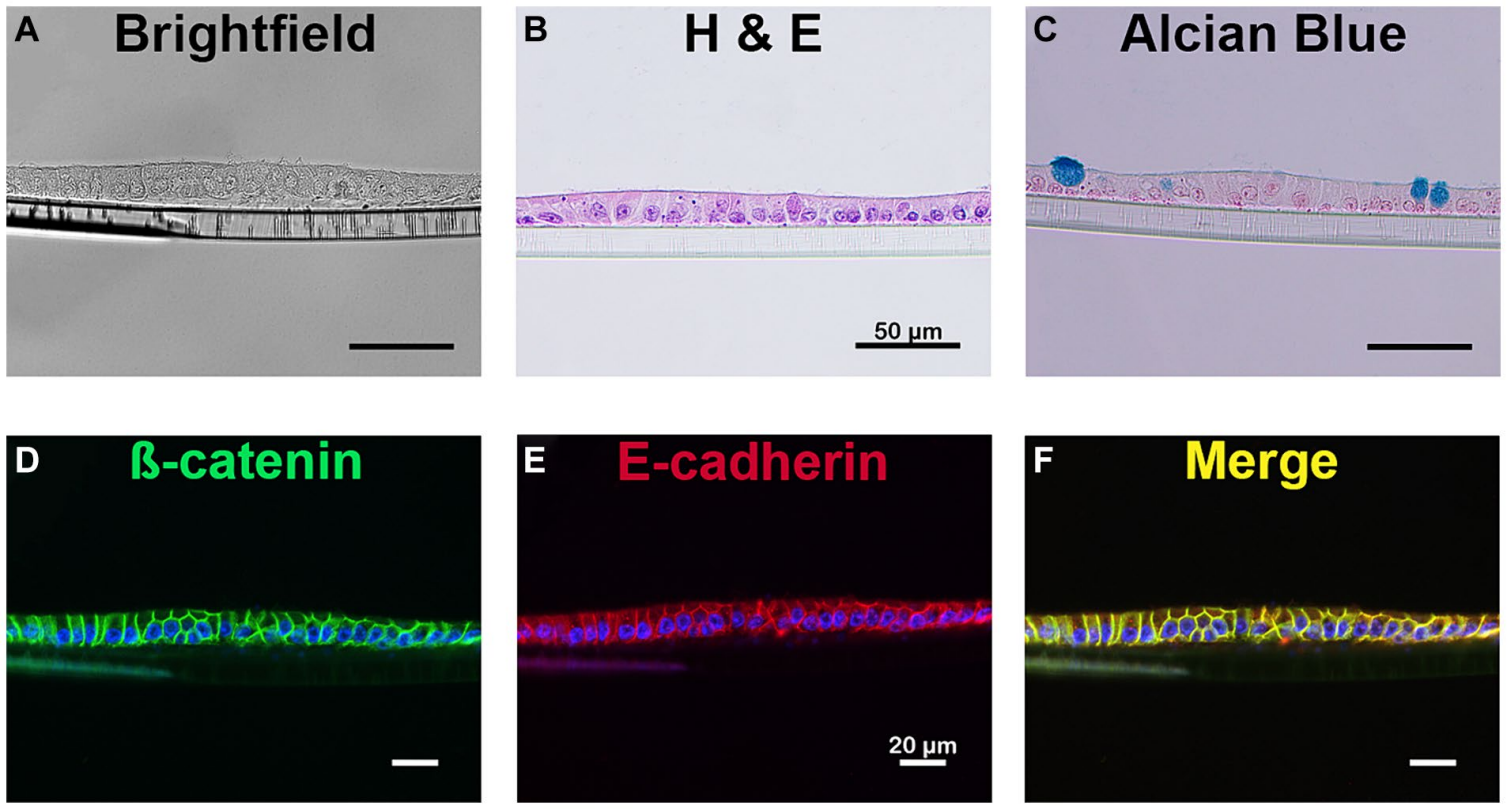
Cellular identification of equine intestinal epithelial monolayer cultures. Mature small intestinal epithelial monolayers were fixed and stained to identify several cell types. Bright field imaging **(A)** and H&E stain **(B)** confirms the presence of a single layer of cells. The presence of mucin producing goblet cells were identified using alcian blue **(C)**. Immunostaining for *β*-catenin (green, **D**) and E-cadherin (red, **E**), membrane-bound proteins, is evident on the cellular border of the monolayer and demonstrates colocalization of the membrane markers (yellow, **F**). Nuclei, blue **(D–F)**. Scale bar 20 μm. *N* = 3.

Following initial plating of dissociated 3D jejunal enteroids (Figure 1B), 100% confluent monolayers were achieved by 5–7 days (Figures 1C, 2).

### 3.2 Presence of epithelial cell lineage biomarkers and tight junction transcripts confirmed using RT-PCR

All assessed gene biomarkers for active stem cells (SOX9, LGR5), reserve stem cells (HOPX, LRIG), epithelial cells (EPCAM), absorptive enterocytes (SIM), goblet cells (MUC2), enteroendocrine cells (CGA), Paneth cells (LYZ), proliferating cells (PCNA), and tight junctions (ZO-1, occludin, claudin-1) were present in jejunum crypts, enteroids and monolayers (Table 4) (17, 27).

**Table 4:** Identification of RNA transcript via RT-PCR in equine jejunum crypts, enteroids and jejunum monolayer cell culture.

Epithelial cell types	Crypts	Enteroids	Monolayer
Active stem cells			
SOX-9	+	+	+
LGR5	+	+	+
Reserve stem cells			
HOPX	+	+	+
LRIG	+	+	+
Epithelial cells—EPCAM	+	+	+
Absorptive enterocytes—SIM	+	+	+
Enteroendocrine cells—CGA	+	+	+
Paneth cells—LYZ	+	+	+
Proliferating cells—PCNA	+	+	+
Tight junction proteins			
ZO-1	+	+	+
Occludin	+	+	+
Claudin-1	+	+	+
Quality control			
β-actin	+	+	+
No template control	−	−	−

+ indicates the presence of corresponding amplicon as assessed via gel electrophoresis, − indicates the absence of a corresponding amplicon.

### 3.3 Two-dimensional monolayer and mucin producing cells demonstrated using alcian blue staining and H&E

Hemotoxylin and eosin (H&E) staining of the equine jejunal monolayer demonstrated a single, two-dimensional monolayer of cells (Figure 2). Alcian blue staining (Figure 2C) identified the presence of a mucin producing monolayer cell types consistent with the presence of goblet cells.

### 3.4 Epithelial markers demonstrated through immunofluorescent antibody staining

Epithelial cells were identified through immunofluorescent antibody staining (28). As demonstrated in Figure 2, the membrane borders of all epithelial cells were positive for both ß-catenin (Figure 2D) and E-cadherin (Figure 2E), two proteins essential in the formation of the epithelial barrier.

### 3.5 Tight junctional complexes, microvilli and goblet cells visualized with transmission electron microscopy

**Figure 3:**
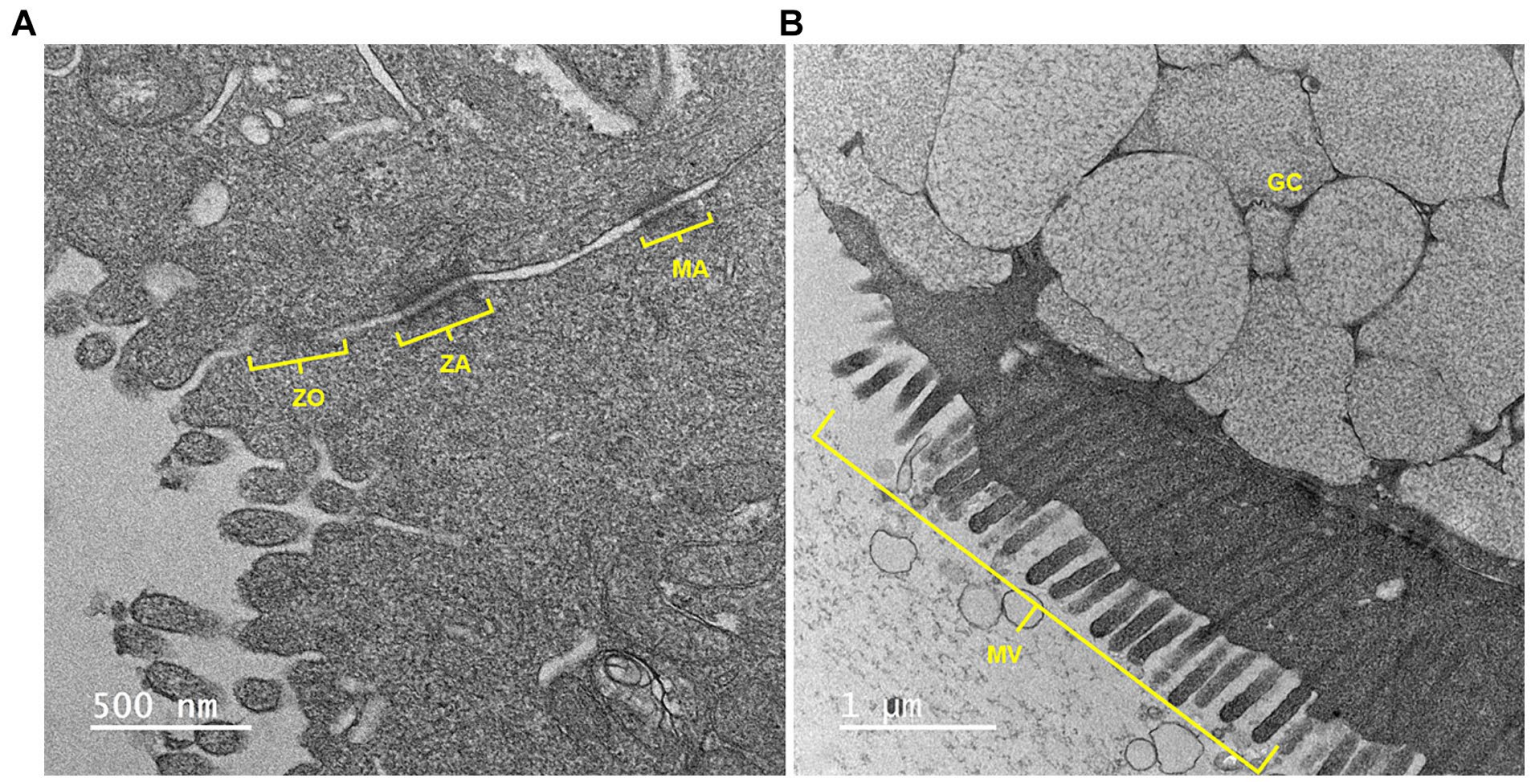
A representative transmission electron microscopy (TEM) image of equine jejunal enteroid-derived monolayers showing **(A)** an intercellular junctional complex and **(B)** a goblet cell with mucin vacuoles. ZO, *zonula occludens* or tight junction; ZA, *zonula adherens*; MA, *macula adherens* or desmosome; GC, goblet cell; MV, microvilli. Scale bar, as indicated.

As shown in Figure 3, TEM demonstrated the presence of the tight junctional complex (zona occludens, adherens and macula adherens), microvilli and goblet cells with mucus-containing vacuoles.

### 3.6 RNA transcripts for tight junction markers visualized within monolayer epithelial cells using RNAscope

**Figure 4:**
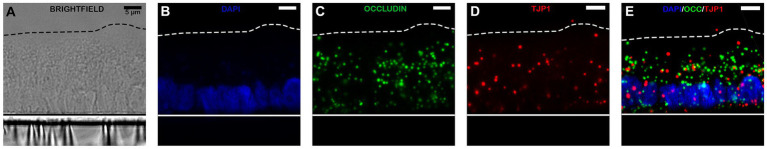
Confocal microscopy images of equine intestinal monolayers expressing tight junction protein (TJP) markers using RNAscope. Markers stained for include DAPI (Nuclei staining; blue, **B**), occludin (OCLN; green, **C**) and tight junction protein/zonula occludens 1 (TJP1/ZO1; red, **D**). Bright-field (BF, **A**) and overlaying fluorescence **(E)** are also shown. The magnification bar represents 5 μm. *N* = 3.

**Figure 5:**
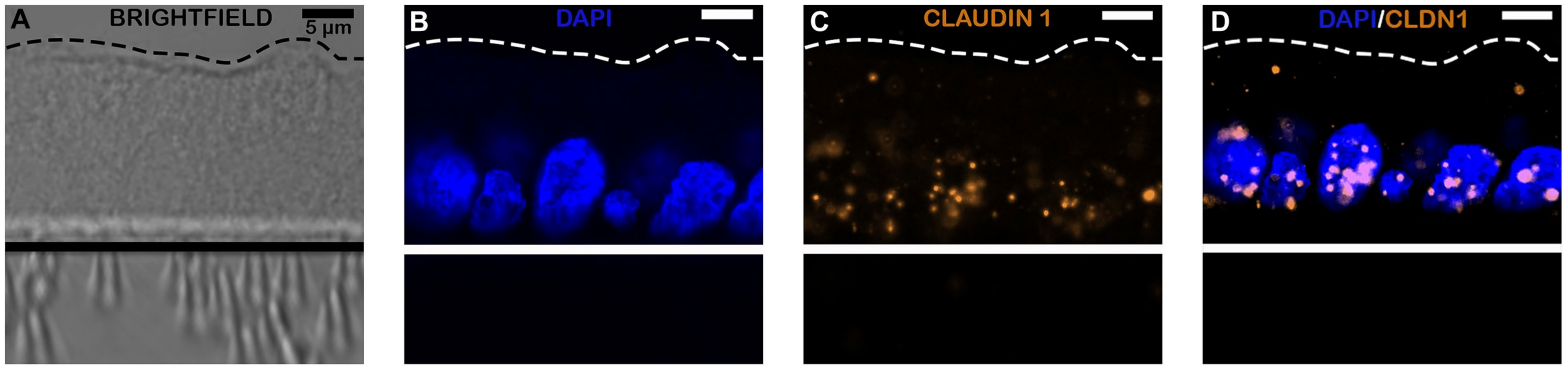
Confocal microscopy images of equine intestinal monolayers expressing tight junction protein (TJP) markers using RNAscope. Markers stained include DAPI (Nuclei staining; blue, **B**) and claudin-1 (CLDN1; orange, **C**), bright-field (BF, **A**); and superimposed-II (overlaying fluorescence: channels 1–5, **D**). The magnification bar represents 10 μm. *N* = 3.

In addition to RT-PCR identification of the presence of both intestinal epithelial cell types and tight junctions, RNAscope was performed to visually assess the spatial location of RNA transcripts within the monolayers. As shown in Figures 4, 5, RNAscope demonstrated the presence of several tight junction markers including tight junction protein (TJP1), claudin (CLD1) and occludin (OCLN) within the cytoplasm of jejunal epithelial cell monolayers.

### 3.7 Measurements of epithelial barrier function obtained using transepithelial resistance

Transepithelial resistance (TER), an indicator of transcellular and paracellular permeability, confirmed the presence of physiologically relevant barrier function of the equine jejunal epithelial monolayers. After 5–7 days of culture, intestinal monolayers had a maximum average TER of 179.9 ohms*cm^2^ ± 33.7 ohms*cm^2^ which is consistent with previously reported *ex vivo* equine jejunum TER (23–25).

**Figure 6:**
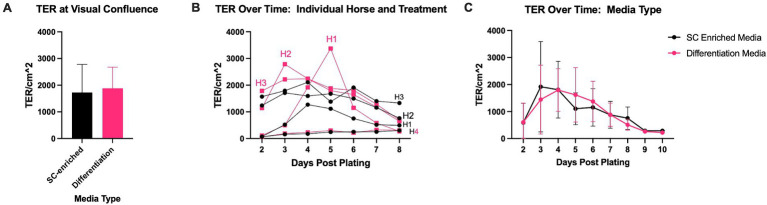
Evaluations of the impact of different media types on TER. TER of monolayers exposed to different types of media was measured upon reaching visual confluence **(A)** and over time. To demonstrate the importance of the individual horse in TER trends, data is represented based upon individual **(B)** and grouped by treatment **(C)**. Statistical significance was determined by two-way ANOVA followed by Tukey’s multiple comparisons, no statistically significant differences were found for any of the comparisons. *N* = 4.

To explore the difference between this TER and those which have been previously published, a separate experiment was conducted to evaluate the impact of different combinations of growth factors, with the potential of driving monolayers to distinct degrees of differentiation, on TER (Figure 6). Within these experiments, TER was highly horse and experiment dependent, though the maximum TER paralleled those previously reported with TER of at least 800 ohms*cm^2^ (18, 27). At confluence, average TER for monolayers treated with a differentiation-media used to promote a mature enterocyte phenotype was higher than monolayers treated with a stem cell-enriching media that promotes immature enterocyte proliferation, though not statistically significant (differentiation media: 1,886 ± 787 ohms*cm^2^ versus stem cell-enriching media: 1,726 ± 1,052 ohms*cm^2^, *p* = 0.74) (Figure 6A). Within this experiment, the TER of monolayers of one replicate from one horse paralleled those seen in the initial experiments (differentiation-media 301.5 ± 22.8 ohms*cm^2^ versus stem cell-enriching media: 245 ± 35.44 ohms*cm^2^) though all others were more similar to previously published TER values (differentiation-media 2,113 ± 494.2 ohms*cm^2^ versus stem cell-enriching media: 1938 ± 934.8 ohms*cm^2^) (Figure 6B). When horses and replicates were grouped by media-type, there was no significant difference in TER over time (Figure 6C).

### 3.8 Physiologically relevant measurements of paracellular permeability obtained with macromolecule flux

Macromolecule flux, an indicator of paracellular permeability, demonstrated minimal macromolecule flux at 30 and 60 min compared to no-cell culture control wells, indicating physiologically relevant paracellular permeability (Table 5).

**Table 5:** FITC-Dextran 400 macromolecule flux.

	Matrigel coated control % (standard deviation)	Equine jejunal epithelial monolayer % (standard deviation)
30 min	98 (13)	17 (7.5)
60 min	99 (0.4)	22 (8.8)

### 3.9 Epithelial restitution times established via scratch assay

**Figure 7:**
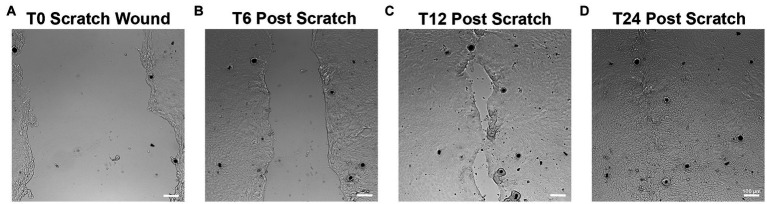
Scratch wound assays to monitor epithelial migration and restitution. After reaching 100% confluency, monolayers were scratched using a P200 pipette tip as shown in **(A)**. Wounds were assessed every 6h for closure **(B–D)**. Epithelial cells can be seen migrating to help close the defect over 12–24 h **(C,D)**. Scale bar = 100 μm. *n* = 3.

All monolayers were successfully scratched using a P200 pipette tip and monitored for wound closure as shown in Figure 7. Visual closure of all scratch wounds occurred within 12–24 h in all horses.

## 4 Discussion

In the present study, a confluent monolayer of the equine jejunum was successfully sub-cultured from enteroids grown from intestinal stem cells (crypts). The cultured monolayer was confirmed to be epithelial in origin via several different methods including RT-PCR for differentiated intestinal epithelial cells, immunofluorescent protein biomarkers and alcian blue staining (mucin producing cells). Evidence of tight junction RNA transcripts was demonstrated by RT-PCR and RNAscope and visualized using TEM. The barrier function and restitution capabilities of the monolayer were assessed via TER, macromolecule flux, and a scratch wound assay. These features support the *in vitro* use of this monolayer culture system to study intestinal barrier function and serve as a model to identify novel equine specific therapies for intestinal barrier dysfunction.

Growth of an equine intestinal monolayer derived from equine enteroids and containing a variety of epithelial cell types confirmed using RT-PCR has previously been reported (18). In the current study, further characterization of these cells was performed using a combination of cell specific staining including immunofluorescent protein biomarkers for epithelial cells and alcian blue staining for mucin. While both studies assess paracellular and transcellular barrier function using TER, we also performed TEM to visualize tight junctional complexes and demonstrated the presence of RNA transcripts for tight junctional complexes by both RT-PCR and RNAscope^®^ and further evaluated paracellular barrier function using macromolecule flux. Of note, some of the TER values in this manuscript differed from those previously reported (18). A wide range of TER values have been reported for monolayer intestinal cell culture including those similar to those reported here (29) and super-physiologic TER measurements (30). Here, we attempted to better understand potential causes for the differences in TER by examining how different growth factor combinations, designed to drive epithelial cultures to varying levels of differentiation, impact TER, as many laboratories utilize differentiation-media for experimentation with monolayers whereas the initial monolayer profiling performed here was performed with stem-cell enriching-media. Additionally, recent work with equine enteroids has demonstrated the potential for different media compositions to influence equine epithelial culture *in vitro* (31). In our media comparisons, no statistically significant differences between the TER of monolayers treated with different growth factor compositions for up to 8 days was found. Additionally, apart from one set of replicates from one horse, the low TER found in earlier experiments could not be consistently reproduced. While each experiment was performed using the same reagents and protocols, they were performed in two different laboratories and using different horses, which may contribute to these variations. Overall, these additional efforts demonstrate the importance of considering individual animal and experiment variability when designing primary cell culture experiments in addition to normalizing each monolayer to itself at baseline to control for such variation. Though our sample size was very small, we did not appreciate any breed or sex-associated trends in our TER values, though none of the horses were stallions so we did not capture that variable. Additionally, all horses used in the TER comparison study were between 10–15 years of age and thus the impact of age could not be assessed. While both manuscripts synergistically advance the study of equine intestinal disease, the present manuscript provides further characterization of epithelial cell differentiation within the monolayer and both tight junction formation and intestinal barrier function and repair.

There are several limitations of this manuscript that should be taken into consideration when extrapolating the results for future studies. Unlike the previously reported study (18), this study did not evaluate the effect of passage on epithelial cell type and barrier function. Ultimately, understanding any potential effect of passage will be imperative in both the long-term use of enteroid derived intestinal epithelial monolayers and interpreting results if studies utilize different passages, but it was beyond the scope of this study. Additionally, there may be horse specific variations based on breed, sex and/or genetic abnormalities that were under or overrepresented in this patient population and should be further evaluated, as suggested by the variation in TER values found in the different experiments performed. Furthermore, this study used commercially available human recombinant growth factors, rather than equine specific growth factors due to their cost effectiveness and availability. Additionally, macromolecule flux was only performed using FITC-Dextran 4000. FITC-Dextran 4000 was chosen as it is a well-studied and accepted probe for evaluating paracellular permeability in cell culture models. But, additional macromolecules such as FITC-LPS or those of different sizes (i.e., polyethylene glycols) could be considered for additional information. Future directions include further characterizing the effect of passage on intestinal epithelial cell types, effect of different culture media and growth factors affecting monolayer growth and behavior, tight junction formation and function and demonstrating the use of the equine intestinal epithelial monolayer in the study of candidate treatments and diseases affecting barrier function in horses.

In summary, here we report the methodology for development of a functional, physiologically relevant equine intestinal monolayer with the ability to advance the study of equine intestinal diseases by providing a characterized platform for *in vitro* work.

## Data Availability

The raw data supporting the conclusions of this article will be made available by the authors, without undue reservation.

## Footnotes

^1^https://acdbio.com
